# Decrease social inequalities return-to-work: development and design of a randomised controlled trial among women with breast cancer

**DOI:** 10.1186/1471-2407-14-267

**Published:** 2014-04-17

**Authors:** Clémence Vidor, Ariane Leroyer, Véronique Christophe, Mélanie Seillier, Jérome Foncel, Justine Van de Maële, Jacques Bonneterre, Sophie Fantoni

**Affiliations:** 1 University Lille Nord de France–Université de Lille III, Unité de Recherche en sciences Cognitives et Affectives (URECA) EA 1059, BP 60149, F-59653 Villeneuve-d’Ascq Cedex, France; 2 SIRIC ONCOLille, F-59000 Lille, France; 3 Caisse d’Assurance Retraite et de Santé au Travail (CARSAT), Nord Picardie-59662, Villeneuve d’Ascq Cedex, France; 4 Centre Oscar Lambret, 59020 Lille Cedex, France; 5 University Lille Nord de France- CRDPD-LEREDS EA 4487, F-59037 Lille, France

**Keywords:** Breast cancer, Return to work, Social inequalities, Intervention, Randomised trial, Psychosocial support

## Abstract

**Background:**

Despite the improvement in the care management, women cancer patients who are still in employment find themselves for the most part obliged to stop working while they are having treatment. Their return-to-work probability is impacted by numerous psychosocial factors. The objective is to describe the development and the content of an intervention aimed to facilitate the return to work of female breast cancer patients and in particular the women in the most precarious situations through early active individualised psychosocial support (APAPI).

**Methods:**

The intervention proposed is made up of 4 interviews with a psychologist at the hospital, distributed over the year according to the diagnosis and conducted on the same day as a conventional follow-up consultation, then a consultation with a specialist job retention physician. We expect, in the first instance, that this intervention will reduce the social inequalities of the return-to-work rate at 12 months. The EPICES score will enable the population to be broken down according to the level of social precariousness. The other expected results are the reduction of the social inequalities in the quality of the return to work at 18 and 24 months and the disparities between the individual and collective resources of the patients. This intervention is assessed in the context of a controlled and randomised multi-centre study. The patients eligible are women aged between 18 and 55 years with a unilateral breast cancer with local extension exclusively, having received surgery followed by adjuvant chemotherapy, in employment at the time of the diagnosis and dealt with by one of the 2 investigating centres.

**Discussion:**

It is essential to assess this type of intervention before envisaging its generalisation. The study set in place will enable us to measure the impact of this intervention aiming to facilitate the return to work of breast cancer patients, in particular for those who suffer from social fragility, compared with the standard care.

## Background

With approximately 53,000 estimated new breast cancer cases in 2011, breast cancer is top of the list of cancers affecting women in France. The improvement in the care therapy and earlier diagnosis are increasing the probability of curing cancer and the 5-year survival rate is above 80% between 15 and 64 years [1]. Even if breast cancer patients have a lower probability of having a job than women in the general population of the same age [2-4], breast cancer often affects working women. The direct consequences of the occurrence of the disease for most of them are the need to take sick leave and the consequent significant loss of earnings [5,6]. Whereas the duration of the sick leave for these women is longer than for those suffering from another type of cancer, the return-to-work rate is higher [7,8]. However, one year after the first surgery, a female breast cancer patient has almost 3 times as much risk of no longer working as a woman in the general population (23% *vs.* 9%) [4].

Return to work is a major issue and frequently affects the quality of life, the financial security, the restoring of a stable social environment and the feeling of normality [9,10]. According to the studies, the return-to-work rates 10 months after the diagnosis vary between 56% and 91% [7,11]. In addition to the factors linked to the treatment and the disease, return to work is impacted by certain psychosocial characteristics. Thus, having a poor level of education, working part-time or experiencing job-linked difficulties (e.g. poor support from colleagues, a big physical and/or psychological workload) accentuate the repercussions of the disease on job retention and their return to work [9,10]. This is why special attention must be paid to the role of the social inequalities in the occupational rehabilitation and reintegration of these women.

In order to mitigate these difficulties and reduce these social inequalities, Amir and Brocky [12] recommend the inclusion as early as possible by the cancer care teams of the return-to-work issue in the course of therapy. The improving of the communication between the various health players, in particular between the general practitioner (GP) and the occupational physician, is also an avenue for intervention to be developed in order to promote a return to work [13]. Several intervention studies concerning the inclusion of actions designed to facilitate return to work in the course of cancer care have been conducted in recent years. However, through a review of the literature, Tamminga *et al. *[3] come to the finding that most of these interventions do not aim in the first instance to improve the return to work, but rather the quality of life and/or the general physical and psychological functioning. Furthermore, these interventions have not for the most part been the subject of randomised trials with a control group, which limits the attribution of the return to work to the intervention [3,14].

Owing to the impact of the factors influencing the return to work, the resultant inequalities and the interventions already conducted, we propose an intervention designed to facilitate the occupational rehabilitation of female breast cancer patients. An early active individualised psychosocial support (APAPI) would enable, in our opinion, help to be provided for the patients facing the greatest social and psychological difficulties and so limit the consequences of their disease on their work and generally speaking improve their quality of life. The objective here is to describe 1) the development and the content of our intervention, 2) the methods used in order to compare the effect of the APAPI on the reduction of the social inequalities of a return to work compared with that of the conventional therapy.

## Methods/Design

### Development of the intervention

The development of our intervention is based at the same time on 1) the factors identified in the literature having a positive or a negative influence on the return to work, 2) the results of the previous intervention studies designed to promote the return to work of cancer patients, 3) the data reported by the « health-employment-information-services » (SEIS) platform of the Lille CHRU (University Hospital Centre), designed to prevent the occupational exclusion of current or previous cancer patients.

### Factors associated with the return to work

Many factors associated with the return to work have been identified in the literature. These various factors can be classified into 3 categories: the individual characteristics, the work-linked factors and the disease or treatment-linked factors.

As regards the individual characteristics, the studies highlight in a consensual way that young breast cancer patients with a higher education level and a high socio-economic level have a more favourable return-to-work prognosis [8-11,15,16]. At the psychological level, a person’s propensity to talk about her disease with her colleagues, to ask for or to receive help and perceive and receive social support from her colleagues and superiors [17,18] and the level of control perceived as high of the disease and the treatment in the workplace are factors linked positively with a return to work [19]. Benefiting from the support of her family and the health professionals, as well as taking care of her health and implementing coping strategies would also facilitate the return to work [20].

As regards the work-linked factors, being in full-time employment at the time of the diagnosis, having a bearable workload, manageable job requirements and a certain job satisfaction appear to be good return-to-work predictors [8,11,19,21,22]. An adjustment of the working conditions at the time of the return to work also helps in bringing the date of this forward [18]. Grunfeld *et al. *[23] also identify that positive representations on the part of the employers concerning the impact of the cancer and the treatment on the work play a role in this process.

Lastly, as regards the factors linked to the disease and the treatment in the return to work of breast cancer patients, Amir and Brocky [12] show that the absence of pain, fatigue, dyspnea, physical limitations, mental co-morbidity, a good previous physical level and good health self-assessment promote the return to work. Furthermore, not having had chemotherapy during the course of treatment also appears to be a factor promoting the return to work [11].

Of the aforementioned individual characteristics and work-linked factors, some reflect a socio-economic precariousness demonstrating the importance of the social inequalities of the return to work: thus low incomes, a greater physical workload and a lower level of education are factors of social inequalities in the return to work for cancer patients compared with their more qualified counterparts with a lower physical workload and better incomes [16,24-26]. Recent studies confirm the impact of social inequalities in rehabilitation programmes for cancer patients. Thus, the study undertaken by Pauwels et al. [27] shows that the younger breast cancer patients with low incomes are, the more they need information and consultations with a psychologist and this right from the onset of the treatment compared with older women with better incomes. As for Holm *et al. *[28] they report that women with a low level of education and low incomes, living on their own, display a greater need for physical, emotional and financial support than women with a higher level of education. The authors recommend moreover paying special attention to people from precarious socio-economic groups, in particular by developing interventions aimed at offsetting these inequalities.

### Results of previous intervention studies

Several reviews of the literature concerning the content of the job-related intervention studies of cancer patients have been conducted by various authors in order to build on the already existing interventions and produce recommendations regarding methods and practices. The review of the literature conducted by Hoving *and al*. [14] is mainly composed of intervention programmes designed to improve physical, psychological and social rehabilitation. Of the interventions included for their methodological qualities, the one performed by Maguire *et al*. [29] shows that the group of patients given the benefit of individual interviews of the « counselling » type by a nurse have a higher return-to-work rate (*n* = 32.76%) than the control group (*n* = 25.54%).

According to Verbeek and Spelten [30], interventions should pay greater attention to and include more information, support and counselling about work-related issues not only from the health professionals but also from the employers. Furthermore, Grunfeld *et al*. [23] recommend the setting in place of a dialogue between employees and employers, as well as adjustments at the workplace in order to meet the needs of the patients and thus enable a gradual return to work.

As for Tamminga *et al*. [3] they put the emphasis on two main return-to-work prognostic factors: self-assessment of work ability and the physical workload. They underline the fact that return-to-work enhancement has rarely been the main objective of a study. More frequently the content of the interventions comprises vocational training, encouragement, work-related counselling and work adjustments.

According to Tiedtke et al. [31], meetings with the family, social and professional environment delivering information about the disease and the treatment would back up the adjustments set in place for breast cancer patients and therefore influence the return to work. However, the communication between the various health professionals is not necessarily efficient and does not provide optimum return-to-work support for the patient [31]. More precisely, the review of the literature done by Boer *et al. *[32] shows that multi-disciplinary interventions have real implications for the improving of the return to work compared with the conventional care model and compared with the other types of intervention.

Tamminga *et al. *[33] continuing the intervention study done by Nieuwenhuijsen *et al. *[13] using a booklet of practical advice (« 10 steps of advice ») for the patient and the occupational physician, propose an intervention study protocol aimed to improve the patient’s return to work via a randomized controlled trial. The content of their intervention is composed of 1) 4 meetings with a nurse in order to start the occupational rehabilitation as early as possible 2) a meeting with the patient, the occupational physician and the line manager in order to draw up a return-to-work plan, and 3) correspondence between the GP and the occupational physician in order to improve communication.

### The « Health-Employment-Information-Services » telephone platform

The « Health-Employment-Information-Services » (SEIS) telephone platform was created in Lille in 2006 given the demand from patients suffering from cancer or chronic diseases, the return-to-work difficulties, the needs highlighted by the professionals, the patients’ ignorance of the many aid facilities in existence and the need to develop local support. The main purpose of this facility is to prevent the occupational exclusion of people suffering from all types of health problems. Nurses trained in listening techniques inform and help the callers with all the procedures of a socio-professional nature. What is involved above all is ensuring the consistency of the support practices between the various health players and anticipating the brakes or the difficulties encountered by the patients with regard to their return to work. When a person calls the platform, they can be given information immediately that is tailored to their situation (pathology, occupational status, health insurance scheme …). Individual contacts with the networks of occupational health, job retention and return to work professionals are also proposed. An individualised follow-up through the periodic calling back of the callers up to 6 months after their enquiry is provided. The SEIS nurses find through their work that both the return to work time and the return to work conditions could be improved if certain steps were taken earlier. This backs up what several studies demonstrate – that job retention or the return to work is conditioned by 1) the support from the work colleagues and superiors for employees, 2) contact between the patient and their company during the sick leave, 3) the anticipating of the pre-return to work examinations with the occupational physician (examinations that take place during the sick leave in order to assess the employee’s state of health and thus facilitate the return to work), 4) the psychological state of the cancer patients and the emotional charge built up by the experience of the disease and the treatment.

To our knowledge no interventions exist that aim more specifically to reduce the social inequalities of the return to work. In the light of these different observations, and taking inspiration from the various results obtained by the intervention studies on the issue of the return to work, in particular the recommendations of Tamminga *and al*. [3], we have formed the hypothesis that the return to work in general and the social inequalities in particular could be positively impacted by the specific support covered by our intervention.

The particular feature of our intervention is the special attention given to the reducing of the social inequalities of the return to work of breast cancer patients; it was therefore this aspect that was emphasised in the construction of our assessment protocol of the effectiveness of this intervention.

### Content of the intervention

These various findings and experiences thus led us to plan the content of our intervention as follows: 1) 4 interviews with a psychologist at the hospital, conducted during the standard course of treatment, on the same day as the conventional follow-up consultation; 2) linked up with a consultation with a specialist return-to-work physician.

### Meetings with the psychologist

Four one-hour interviews are scheduled throughout the course of treatment. These interviews are coupled with the necessary travel for the dispensing of the care and therapy, not generating therefore any additional travel for the patients. These interviews are based on the problem solving method [34].

The first interview is conducted at the start of the treatment, on the same day as the first chemotherapy session. Its purpose, through a semi-directive interview, is to identify the repercussions of the disease and the treatment on the patient’s job situation, clarify her needs and her expectations with regard to her job, and her wishes and difficulties with regard to this situation.

The second interview is conducted on the same day as the 6^th^ chemotherapy session. Its aim is to help the patient clarify the gap between the actual situation and the desired situation thanks to problem analysing techniques. The situations to be overcome are analysed with precision, as are the brakes and the resources thanks to an analysis of the psychological tests that the patient will have undergone during the pre-test (detailed below). This intervention positions these psychosocial tests both at the level of the overall assessment of the research project and at the level of the intervention itself as a tool helping the patients to become aware of their resources, difficulties and changes over the course of time. With the patient’s agreement, an area for work resulting from the questionnaires is chosen (social support, coping, attitude with regard to problems). The SEIS platform is presented as a practical tool that can be mobilised during this interview in order to anticipate the return-to-work strategies as early as possible.

The third interview is conducted during the final chemotherapy session. It invites the patient to invent new strategies for reducing the difficulties identified via problem-solving techniques. The psychologist helps the patient to develop her psychosocial skills, test out new behaviours and call upon the external resources available such as the SEIS platform in order to promote the return to work. Depending upon the problem to be solved, patients may be offered relaxation techniques, role playing, or contacts with local professionals.

Between the 3^rd^ and the 4^th^ interview, the patient is invited to choose new strategies she feels capable of implementing in order to test them out in a real situation. This step corresponds to the implementation of an action plan.

Finally, the fourth interview is conducted 3 months after the completion of the radiotherapy, at the same time as a post-radiotherapy follow-up consultation. Its purpose is to examine one by one the adaptation strategies that have been tested between sessions 3 and 4 in order to determine their advantages and drawbacks, the results obtained and the emotions felt. It is a question of strengthening the feeling of self-efficacy thanks to the objectives attained, pursuing the problem-solving learning process with the objectives that have not been attained, generalising the new effective adaptation strategies and rethinking the ineffective adaptation strategies.

### Meeting with the job retention specialist physician

Support from the psychologist is complemented, following the final interview, with a job retention/return to work consultation with a physician specialising in the return to work. To this end the patient is asked to come with her latest medical results to a medical consultation at the usual treatment site. The return-to-work specialist then drafts a summary, using as a basis to work from the medical file and the psychosocial support assessment, together with recommendations for a return-to-work plan. This summary is forwarded, via the patient, to her company’s occupational physician and her GP. The aim of this consultation is thus to facilitate the communication between the various players involved in the return to work of the patient and promote a multi-disciplinary analysis of the patient’s needs and expectations.

### Intervention assessment methods

In order to assess the effect of this early active individualised psychosocial support (APAPI) compared with the conventional treatment of the reduction of the social inequalities of the return to work at 12 months, we set in place a comparative, randomised, prospective and multicentre intervention study.

The secondary objectives were to assess the effect of this APAPI compared with the conventional treatment on the quality of the return to work at 18 and 24 months, and on the individual and collective resources of the patients such as the perceived social support, the coping strategies, the attitude to problems, anxiety and depression, the quality of life and the contacts with the SEIS platform. Lastly, a cost analysis of the benefit of this action is also planned.

The Committee of Protect People NORTHWEST III gave a favorable opinion for the study APAPI the September 8, 2012. The CPP reference for this study is: 2012-13.

### Participants

In order to carry out this study we opted for the population of women aged between 18 and 55 years with a unilateral breast cancer with local extension having been given surgery followed by adjuvant chemotherapy, with or without radiotherapy, in employment (salaried employees, trades people and the professions) at the time of the diagnosis and dealt with by one of the 2 investigating centres.

The criteria for non-inclusion are the presence of in situ lesions, cancer relapse, a metastatic cancer at the outset, a bilateral location. The patients with physical, psychic, psychiatric or cognitive disabilities preventing them from replying to the questionnaires and taking part in the interviews, and people having received prior treatment for a serious illness lasting at least 6 months (owing to another pathology) and people under wardship or guardianship are also not included in this study.

### Recruitment and investigating sites

In order to have the most representative population possible, the recruitment and the intervention are performed at two investigating centres in Nord-Pas-de-Calais, the Lille Centre Régional de Lutte contre le Cancer (Centre Oscar Lambret) and the Douai Centre d’Imagerie et de Cancérologie (Centre Léonard de Vinci).

The Centre Oscar Lambret is a Private Health Establishment of Collective Interest highly specialised in cancer research. It is associated with the Lille University Hospital Centre (CHRU) and is part of the group of Cancer Centres within the French Federation of Cancer Centres. By belonging to this group, the Centre Oscar Lambret provides an additional guarantee of quality and effectiveness to its cancer treatment, teaching and research actions at the service of the patient. The centre deals with 1,800 to 1,900 breast cancer patients per annum, i.e. 30 to 35% of the cases in the region.

The Centre d’imagerie et de cancérologie Léonard de Vinci is a private establishment associated with a medical group comprising radiology, radiotherapy, scanner and MRI centres.

The centre treats 1200 to 1300 new cancer cases every year (all locations taken together).

### Care and randomisation of patients

The standard care for breast cancer patients at each site is the same: following the cancer diagnosis, the medical files of the patients are examined at a multidisciplinary coordination meeting the purpose of which is to decide on the course of treatment to be given. Within a timescale of 1 to 2 weeks from the diagnosis, the patients are seen by an oncologist who explains to them what their course of medical treatment will consist of and whether or not this will involve chemotherapy. An appointment is then made for the following week with a nurse for a pre-chemotherapy consultation. During this interview lasting approximately 90 minutes, the nurse reviews with the patient what she has understood about the diagnosis and the treatment and her concerns and needs. The nurse can direct the patient to other professionals (psychologist, dietician, physiotherapist, welfare worker). The first chemotherapy session takes place approximately 1 month after this consultation.

The study is therefore proposed by the medical oncologist during the first care consultation to the patients fulfilling the inclusion criteria in the 2 investigating centres. After having explained the study to the patients with the information leaflet, those who accept sign a consent form. Next the patients will be randomised into two groups: 1) Group A: the « intervention » group which will have the benefit of the early active individualised psychosocial support in addition to the usual care. 2) Group B: the «standard care » group hereafter referred to as the “control group” which will follow the regular course of treatment without having the benefit of the psychosocial support as described above. This standard care already includes tools for identifying situations of great social distress enabling the patients to be directed to the regular social helpers.

Just after the inclusion, and before the start of their chemotherapy treatment, both groups of patients undergo the same battery of tests (pre-test).

**Figure 1:**
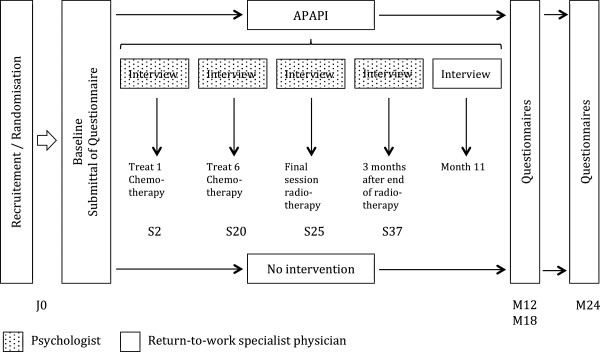
Study design.

They are then invited to do the same battery of tests and fill in the questionnaires again by letter 12 months after the start of their chemotherapy treatment. Follow-up of the return to work in terms of the rate and the conditions and a follow-up of the quality of life are also done at 18 and 24 months. In the event of their failure to reply to the questionnaires, the patients are re-contacted twice by telephone within the space of 15 days following the sending out of the questionnaires (Figure 1).

### Questionnaires and psychological tests

During the pre- and post-tests (on the inclusion and 12 months after the diagnosis), for the two groups (A « intervention » and B « control »), we use a battery of tests with a questionnaire and validated psychological tests.

The questionnaire covers the socio-demographic characteristics of the patients, their disease (objective and subjective criteria such as fatigue, anxiety, mood disorders…), changes in their socio-professional situation during the study, as well as the difficulties they encounter with regard to the repercussions of their cancer on their situation (sick leave, financial aid, employment, recognition of disability, psychological aid, paramedical assistance and guidance). Social fragility is estimated using the Epices score [35], which will enable the women who are the most fragile socially speaking (FS+) to be distinguished from those who are less fragile socially (FS-) through a cut-off at the median. The Epices score was in fact built on a questionnaire comprising the various dimensions of precariousness: nationality, level of education, occupation, composition of household, housing, social protection, perceived health, income, leisure activities, financial difficulties, social integration, seeking healthcare, serious events experienced before the age of 18 years.

The measurement of the social support is done by the QSSP [36], the coping strategies of the patient by the WCC [37], the attitude to problems by the QAP [38], anxiety and depression by the HADS scale [39] and the quality of life by the FACT-G [40]. The quality of the return to work is estimated by the assessment of the work ability (WAI) [41].

### Sample size

The number of patients was defined according to the expected reduction in the social inequalities of the return-to-work rate.

The research done by Quinton-Fantoni *et al. *[9] showed that the return-to-work rate 12 months after the diagnosis of women having had chemotherapy was 50% on average, but there was a difference of 22% between the most precarious and the least precarious cases.

The recruitment of 125 patients per group (A « intervention » and B « control »), with the acceptance of a first order risk of 5% and a power of 80%, would enable a reduction in the gap between the precarious cases and the non-precarious cases of 13.5 points to be demonstrated, enabling the difference in the return-to-work rate to be lowered from 22% to 8.5%. In order to make allowance for possible drop-outs during the study and unusable data, the minimum number of patients to be recruited was increased by 20%. The final number therefore comes to 150 women per group, i.e. a total of 300 patients.

### Data management

A dedicated database was created for the study which was tested out and validated before any data was entered. This dedicated database will be developed with the aid of Capture System (CLINSIGHT), which is a software package designed for the global management of clinical studies and meeting the study’s regulatory requirements. A data validation plan will be drawn up and will describe in detail the checks to be performed for each variable together with the list of the blatant errors for which correction is authorised.

The observation books will be checked when they are collected and then the data will be entered followed by post-checking (data entered/hard copy data). This data will be checked once again by the team in charge of the data management via the error messages received from the validation programmes. Blatant errors will be corrected. The other errors, omissions or inconsistencies will be mentioned on correction request forms that will be sent to the investigating physician and the corrections included in the database.

The database will be frozen after the final quality control then exported to the SPSS statistical software as per an automated and validated procedure.

### The participants

In the context of the study, two psychologists are responsible for doing the APAPI, each one reporting to an investigating site. In order to provide similar support from a theoretical and practical standpoint, and in accordance with the recommendations of Tamminga et al. [3] who stress the importance of individual intervention with a cognitive behavioural component, both the psychologists received prior training in cognitive behavioural therapy and work with an interview template specific to each meeting with the participants. Regular meetings will be held in order to harmonise their practices during the support provided.

The occupational physician of the occupational diseases and job retention unit of the Lille CHRU, a return-to-work specialist, is responsible for receiving each participant of the study following the 4 interviews with the psychologists. These consultations take place at the hospital department administering the standard care.

### Results expected

This is a multi-disciplinary project through which we hope the APAPI will enable a reduction in the social inequalities of the return to work (timescale, conditions, job retention rate) of the breast cancer patients compared with those who do not have the benefit of the APAPI.

The main observation we expect to make is that the social inequalities of the return-to-work rate 12 months after the diagnosis of the breast cancer patients are less for the APAPI group than for the control group.

We also expect to observe that this reduction in the inequalities in the intervention group compared with the control group also concerns the conditions and the return to work at 12, 18 and 24 months after the diagnosis, the psycho-social factors studied and worked on with the patients, that quality of life of the patients at 12, 18 and 24 months and the proportion of people having made use of the SEIS platform.

Another result expected is the overall improvement in all these factors in the intervention group compared with the control group.

### Statistical analyses

The data will be entered twice then exploited in SAS V9.2 and SPSS v20. After the quality of the data has been checked and any corrections made, a description of the population included in the study will be given. A comparison of the main return-to-work criteria of the two groups drawn at random will be done (age, stage of development of the disease, occupational category).

The reduction in the social inequalities of the return to work will be studied by comparing the deltas (difference in the return-to-work rates between the two sub-groups FS + and FS-) between the control group « B » and the intervention group« A » having the benefit of the APAPI. A rough comparison of the two deltas will be done with a Chi2 test. The taking into account of adjustment factors (age, secondary effects of the treatment …) will be done with the aid of multivariate regression models.

As regards the secondary objectives, what is involved here is comparing for the 2 groups:

1) the deltas of the quality of the return to work at 18 and 24 months after the diagnosis (WAI and job retention rate); 2) the deltas of the psychosocial characteristics (QSSP, WCC, QAP, HADS and FACT-G scores) and the external resources (number of contacts with the SEIS platform) at 12 months from the diagnosis.

### Medico-economic analyses

From the standpoint of the cost-benefit analysis, the APAPI support action protocol will be useful to implement compared with the conventional support action if the additional benefits are greater than the additional costs.

The point of departure for the analysis is situated in the identification of the mean marginal effect (in the population treated) of the support action on the probability of regaining employment after a certain fixed period of time (or over the time actually required itself). This effect is measured by the statistical model and provides the weighting for the various costs and benefits linked to this action.

The social security schemes and the company employing the person are the main beneficiaries of the early return to work through the number of days of sick leave saved and the rise in productivity due to the return to work of a person *in theory* better qualified than her replacement. On the markets where there is unemployment, the benefit will have to be reduced by the amount of the unemployment benefit paid to the replacement.

The monetary costs result mainly from the additional expenses incurred by the APAPI measure. They include the intervention of the psychologists and the investigating teams; it is then a question of calculating the net additional cost. It is however necessary to take into account the opportunity costs of the staff used for the intervention as they would be employed in other tasks if they were not involved in the APAPI measure. The monetary costs also include the fixed operating costs (facilities, equipment, expenses, etc.) and the cost of the increase in the activity of the SEIS platform.

*Ultimately*, several scenarios will be envisaged depending upon the elements that it is decided to include in the costs and benefits: various situations will be tested out to give a broad range of possibilities for the public decision-makers when making their assessment of the APAPI measure.

## Discussion

The originality of our study resides in the specific nature of our intervention. In point of fact, few interventions aim to reduce the social inequalities of the return to work of breast cancer patients.

Consistently with the previous research [3,27] our protocol is included in the existing course of treatment as early as possible so as not to make the patient support too unwieldy. The meetings with the psychologist therefore take place where the standard care is administered. Next, the inclusion of the SEIS platform enables interactive and personalised support to be offered to the participants that is directly linked to their work-related questions, which is different from most of the studies which supply an educational leaflet [13,33]. Lastly, in our intervention, the meetings are conducted by psychologists trained in cognitive behavioural therapies, enabling the psychosocial skills of the participants to be developed that relate to the problem solving techniques.

At the methods level, the purpose of our study is to develop an intervention that is assessed in the context of a randomised controlled trial, which constitutes the first strong point of our research. We have in fact taken into account the recommendations resulting from the previous studies deemed sometimes as weak from the methods standpoint, in particular because of the absence of a control group. The second strong point of the study resides in the participation of two of the region’s investigating centres in order to increase the heterogeneousness of the population studied. In point of fact each investigating site uses different socio-demographic and economic criteria for the investigation of its population. The Centre Oscar Lambret, located in the heart of the Lille metropolitan area, deals rather with patients from the tertiary sector, whereas the Centre Léonard de Vinci in Douai, which is located rather in an industrial and rural area, deals with the patients from primary and secondary sectors. Randomisation is therefore done independently at each investigating site in order to best preserve the socio-demographic criteria that will enable the social inequalities of the return to work to be observed.

The third strong point concerns the recruitment of the participants. In order to limit the biases, we chose to select women aged between 18 and 55 years; in point of fact beyond the age of 55 the French social security system proposes specific care packages enabling patients close to the retiring age not to return to work. In the light of the results of the literature on the impact of chemotherapy on the return-to-work time [11], we also opted to exclude any patient not given adjuvant chemotherapy.

In theory our study is likely to be inherently limited by the very construction of the protocol. In point of fact, given that the two investigating sites are located in the same region, there is a limit on the generalising of the results of the efficacy of this protocol at the national and even international level. What is more, APAPI is based on the SEIS platform which is not a standardised service in France. Thus the setting in place as it is of this intervention in other regions would require alternatives in terms of interactive individualised support equivalent to the SEIS platform. Moreover, in the light of the statistics, Nord-Pas-de-Calais is one of the regions in France most affected by cancer and unemployment. So, if the intervention successfully impacts the social inequalities of the return to work in our region, we consider we are justified in thinking that a similar scheme to APAPI would also act on these inequalities in other regions, provided the planned organisation is kept to: conducting of the interviews with the psychologist not requiring any additional travel on the part of the patients, conducting of the interviews with the physician specialising in job retention at the site where the patients are treated, communication by letter between the physician specialising in job retention, the GP and the occupational physician.

### Clinical prospects

The data collated from the A « intervention » and B « control » groups will enable the difficulties and socio-professional inequalities usually encountered by breast cancer patients to be identified, as well as the medical, social, psychological and economic determinants.

The identification of the psycho-socio-economic determinants as return-to-work factors would enable optimisation of the support for breast cancer patients. The generalising of support practices on the basis of the relevant factors holds out the prospect of a positive economic impact and above all better return-to-work conditions for the people having been treated for cancer with the aim of maintaining an acceptable quality of life for them, creating a dynamic and positive repercussions on everything that may have been affected by the cancer and its treatment (sphere of social, professional and personal life, psycho-social skills).

The aim of this support being to take into account and modify certain factors of psychosocial fragility (poor perceived social support, little in the way of developed coping strategies, attitude to negative problems), we expect to observe a benefit for all the patients but a greater one for those in a situation of social fragility.

We hope to test out an original intervention that offers, not the same thing for everyone, but more for those who are in greater need than the others, so as to reduce the dual and even triple distress when the sick person is in a situation of social, psychological and/or occupational fragility.

## Abbreviations

APAPI: Early active individualised psychosocial support; EPICES: Evaluation de la précarité et des inégalités de santé pour les centres d’examens de santé; GP: General practioner; SEIS: Health-employment-information-services; CHRU: University hospital centre; QSSP: Questionnaire de soutien social perçu; WCC: Ways of coping check list; QAP: Questionnaire d’attitude face aux problèmes; HADS: Hospital anxiety and depression scale; FACT-G: Functionnal assessment of cancer therapy - General; WAI: Work ability index.

## Competing interests

The authors declare that they have no competing interests.

## Authors’ contributions

VC, SF, AL, JF and CV have been involved in drafting the manuscript; JF and AL was the statistical advisor; CV, AL, VC, MS, JF, JB and SF have been involved in the study conception and design, assisted in writing the manuscript and have given final approval of the version to be published. All authors read and approved the final manuscript.

## Authors’ information

SF is the study coordinator, obtained the grant and is responsible for the present paper, JB and VC are the coo-coordinators of the study.

## Pre-publication history

The pre-publication history for this paper can be accessed here:

http://www.biomedcentral.com/1471-2407/14/267/prepub
